# Ragu: A Free Tool for the Analysis of EEG and MEG Event-Related Scalp Field Data Using Global Randomization Statistics

**DOI:** 10.1155/2011/938925

**Published:** 2011-02-20

**Authors:** Thomas Koenig, Mara Kottlow, Maria Stein, Lester Melie-García

**Affiliations:** ^1^Department of Psychiatric Neurophysiology, University Hospital of Psychiatry Bern, University of Bern, 3000 Bern 60, Bolligenstr. 111, Switzerland; ^2^Neuroinformatics Department, Cuban Neuroscience Center, Havana 15202, Cuba

## Abstract

We present a program (Ragu; Randomization Graphical User interface) for statistical analyses of multichannel event-related EEG and MEG experiments. Based on measures of scalp field differences including all sensors, and using powerful, assumption-free randomization statistics, the program yields robust, physiologically meaningful conclusions based on the entire, untransformed, and unbiased set of measurements. Ragu accommodates up to two within-subject factors and one between-subject factor with multiple levels each. Significance is computed as function of time and can be controlled for type II errors with overall analyses. Results are displayed in an intuitive visual interface that allows further exploration of the findings. A sample analysis of an ERP experiment illustrates the different possibilities offered by Ragu. The aim of Ragu is to maximize statistical power while minimizing the need for a-priori choices of models and parameters (like inverse models or sensors of interest) that interact with and bias statistics.

## 1. Introduction

Scalp field measurements represent activity of electrically active extended neural generators in the brain and offer a unique window to measure human information processing noninvasively and with a high time resolution. Today, EEG and MEG recording systems can record human scalp field data with high density in space (>100 sensors) and time (>1000 Hz), which improves the resolution of the results. However, the understanding of effects observed on the scalp has been severely hindered by the so-called inverse problem of EEG and MEG measurements, which prevents in the general case that effects observed on the scalp can be unambiguously attributed to a specific set of brain tissue. As a consequence, many of the results found in the literature depend at some point on some implicit or explicit model, and since these models vary considerably, unambiguous conclusions across studies and models are often difficult to draw. 

The aim of the current paper is to present methods and software that allow users to analyze event-related scalp field data using methods that incorporate the physical underpinnings of scalp electromagnetic data but are model independent. The software should enable researchers to assess the significance of ERP effects globally, and without need of a-priori assumptions about the correct model, (i.e., about the location of “active” or “inactive” sensors, or about the correct parameters for a source model). Evidence for an effect based on such unbiased statistics can then entail further more model-based analyses implemented in other tools. In the remainder of the paper, we will develop the methodological background of the procedure, followed by a brief description of the software implementation, a sample analysis to illustrate the procedure, and a discussion of the implications.

## 2. Methodological Background

The physics that relates the intracranial brain-electromagnetic activity to the extracranial sensors is summarized by the so-called leadfield or forward solution of the EEG/MEG [1]. The leadfield of the EEG and MEG defines weighting factors that linearly translate the activity of a given source to scalp potential differences; these weighting factors depend on the sensors position, the location and orientation of the source, and eventually (for EEG data) on the geometry and electromagnetic properties (i.e., conductivity, homogeneity) of the different tissues between source and sensor. The leadfield is a smoothing operator that blurs the measurements in space [1] and introduces correlations among the sensors depending (among other factors) on the distance between them. As a result, there are three important facts to take into account when dealing with EEG/MEG scalp field data:

1. the activity of even a point-like source will produce a field that extends across the entire scalp, such that most sensors will pick up a signal from that source;
2. a single sensor can pick up signals from many different and eventually remote sources;
3. for the case of EEG, since all measurements are potential differences, the signals recorded at a given electrode are always dependent on, at some point, an arbitrary choice of reference.

In our opinion, a major part of the publications that have employed scalp potentials to investigate the effects of some experimental manipulations have not taken these facts sufficiently into account, such that the interpretability of the obtained results is seriously limited. As a consequence, the impact of ERP studies is probably below the original potential of the measured data.

### 2.1. Statistical Assessment of Topographic Effects for One Time Point

In general, the aim of a statistical comparison of scalp field maps between two or more conditions at a given time point is to test whether some of these conditions consistently differed in active sources. Interestingly, such arguments can be made without estimating the location of those sources. This is so because scalp fields are additive; if two sources are active at the same moment in time, the data measured is the sum of the two scalp fields produced by the two sources. This implies that we can also interpret the difference of scalp fields observed during different conditions. This difference scalp field is identical to the scalp field of those sources that were different between the two conditions. (All sources that were identical in the two conditions cancel out when the difference is computed). 

In order to test whether some conditions differ in active sources, it is thus sufficient to show that there are scalp field differences between these conditions, and that these differences are unlikely to have occurred by chance. To avoid any biases, this evidence can be based on quantifying the overall amount of difference of activity, that is the overall strength of scalp field differences. Once such a quantifier is available, it can be used to test the measurements against the null hypothesis. The suggested quantifier and the suggested statistical testing rely on previously reviewed and published papers [2, 4, 5] and are briefly explained below. 

A global and well-established quantifier of scalp field strength is the Global Field Power (GFP, [6, 7]). As shown in formula (1), the computation of GFP is analogous to the computation of the standard deviation across all sensors

(1) $$\text{GFP}=\sqrt{\frac{\sum_{j=1}^{n}\left(v_{j}-\bar{v}\right)}{n}},$$

where *v*_*j*_ is the voltage measured at sensor *j*, *n* is the number of sensors, and $\bar{v}$ is the mean measurement across all sensors. The GFP can be shown to be reference independent. Given that the sensor array covered a sufficient part of the scalp, using the GFP of scalp field differences to quantify the effect of an experimental manipulation is thus compatible with the three important facts about EEG/MEG scalp data mentioned in the introduction:

- since all sensors are taken into account, the scalp field produced by difference source(s) is taken into account to its largest possible extend (no problem with fact 1);
- since all sensors are being used, false negatives based on partially overlapping scalp fields are unlikely (no problem with fact 2);
- there is independence of the reference (no problem with fact 3).

As previously outlined [3], the usage of the GFP of a difference map can be generalized to cases with more than two conditions and/or two or more groups using a global measure *s* of scalp field differences as defined below

(2) $$s=\overset{c}{\underset{i=1}{\sum }}\sqrt{\frac{\sum_{j=1}^{n}{\left({\bar{v}}_{ij}-{\bar{\bar{v}}}_{j}\right)}^{2}}{n}},$$

where *c* is the number of conditions and group, *n* is the number of sensors, $\bar{v_{ij}}$ is the voltage of the grand mean across subjects of condition and/or group *i* at sensor *j*, and ${\bar{\bar{v}}}_{j}$ is the grand mean across subjects and conditions of the voltage at sensor *j*. All data has to be against the average reference [3].

If instead of a group/condition membership a predictor is available that is assumed to be linearly related to the activity of an unknown set of sources, the scalp field produced by this set of sources can be estimated using the so-called covariance maps *β*_*j*_ [4]. Given a set of scalp field maps *v*_*ij*_, where *i* is the index of the observation and *j* is the sensor, and given for each map *v*_*ij*_ the predictor *b*_*i*_, the covariance map of *v*_*ij*_ and *b*_*i*_ is given as follows:

(3) $$\beta_{j}=\overset{m}{\underset{i=1}{\sum }}v_{ij}\cdot b_{i}.$$

As mentioned above, the quantification of the overall strength of the sources that account for the predictor *b*_*i*_, the GFP of the covariance map can be used. In this case, *s* is defined as

(4) $$s=\sqrt{\frac{\sum_{j=1}^{n}{\left(\beta_{j}-\bar{\beta }\right)}^{2}}{n}},\;\;\text{where}\;\bar{\beta }=\overset{n}{\underset{j=1}{\sum }}\beta_{j}.$$

As argued in previous papers [3], the value of *s* depends on the amplitudes of the mean differences among conditions and/or groups, and on some random variance across subjects and conditions. For the assessment of the significance of an effect, we are interested in whether the value of *s* is solely due to random variance across conditions and/or groups, or whether it is at least partially caused by a certain consistency of an effect across the measurements obtained in the different groups and/or conditions. This can be tested by randomly shuffling the group and/or condition assignments in each subject and recompute *s*. The resulting value of *s* will then depend only on the random variance across subjects and conditions, but an eventual consistency of differences among groups and/or conditions across subjects (i.e., an effect of group and/or condition) has been eliminated by the randomization procedure. Any value of *s* obtained after random shuffling is thus an instance of *s* under the null hypothesis, stating that some differences are due to noise alone. By repeating the random shuffling and computation of *s* many times, one can obtain an estimate of the distribution of *s* under the null hypothesis and compare the value of *s* in the real data against this distribution. The significance of the effect, that is the probability of the null hypothesis, is then given by the percent of randomly obtained values of *s* that are larger than or equal to the value of *s* obtained with the real data. In the literature, the procedure to compare groups and/or conditions has been called TANOVA (topographic analysis of variance); if a linear predictor is used, the proposed term is TANCOVA (topographic analysis of covariance). 

In general, nonparametric randomization statistics as those described above are known to have similar statistical power as classical parametric tests if the assumptions made by the parametric tests hold, and have better power otherwise [8]. One important additional point has, however, to be taken into account when applying randomization statistics, which is exchangeability. Exchangeability means that the distribution of the effect sizes remains the same after the shuffling. This may become a problem in fMRI data, where the spectrum of the physiological data is at or below the spectrum of the experimental design [9]; for EEG and MEG, this is, however, not a problem, because the events to be analyzed are typically very short, and instantaneously measurable at the sensor level. Furthermore, it is obvious that the number of observations sets a limit to the number of possible permutations, which set a natural lower limit on the possible level of significance.

### 2.2. Statistical Assessment of Significance across a Time Interval

In ERP experiments, it is often not a priori clear at what latency window an effect can be expected, and the analysis needs to explore the data across many time frames. This may obviously inflate the possibility of false positive findings due to multiple testing, and some test for the overall significance of an effect is necessary. In previous papers [2, 3], we have proposed to obtain such indices of overall significance by estimating how likely it was that the overall count of significant time points (at a given threshold of significance) could have been observed by chance, or how likely it was that the observed duration of a period of significant effects would have been observed by chance. 

If randomization statistics have been computed, such overall statistics can be directly derived from a further analysis of the results of the randomization runs. Following the description in Koenig and Melie-Garcia 2010, we illustrate the procedure for the overall count of significant time periods. First, a threshold for significance is chosen, and the count of the number of significant time points in the data is established, which will serve as the overall measure of effect size. As before, this effect size needs to be compared to the distribution of the count of false positives assumed to occur under the null hypothesis. 

In the present case, we can estimate the distribution of the count of false positives from the randomization runs. We assess, for each randomization run *r* and time point *t*, a “pseudo-*P*-value” *P*′ defined as the percentage of cases where the measure *s* of *r* was larger than the measures *s* obtained in the remaining randomization runs. For a given randomization run *r*, we thus obtain *P*′ values at each moment *t*, and we can establish the count of *P*′ values that are lower than the above chosen threshold of significance. This count is thus an instance of the number of time points with *P* values below the critical threshold while the null hypothesis still holds on a global level. If this count of false positives is assessed for all randomization runs, an estimate of its distribution under the null hypothesis is obtained. The count of significant time points obtained in the original data is then compared against this distribution, yielding an overall estimate of the significance of the difference. This test is similar to cluster-size statistics used in MRI data analysis and have been described elsewhere [9].

For the assessment of significance of the duration of an effect, the analogous procedure can easily be inferred.

### 2.3. Data Normalization

For the interpretation of significant differences between two or more scalp fields, it is sometimes useful to make a distinction between two specific cases. In one case, the distribution of the active intracranial sources is the same in all conditions, and the differences among conditions can be explained by a scaling factor that is common for all these active sources. Functionally, one would thus interpret such a difference as a quantitative difference of activation in the presence of apparently similar brain functions. Because of the above-outlined linear relation between intracerebral sources and scalp field measurements, the same argument can be made if the measured scalp fields differ merely by a scaling factor that is common for all sensors. If (and this is the alternative case) differences between scalp fields cannot be solely explained by a scaling factor common for all sensors, the active intracerebral sources must have had at least a partially different location and/or orientation, which can be considered as a qualitative difference and indicates that at least partially different brain functions have been recruited. In order to distinguish these two cases, the program offers the possibility to normalize the variance of the scalp fields across sensors before the statistical tests are computed. This eliminates the effect of potential differences in scaling, such that significant results obtained with normalized data can be taken as evidence of qualitative difference, or evidence for the recruitment of at least partially different brain functions. Therefore, to complete an analysis that was based on normalized data, it is thus suggested to run separate univariate statistics on the spatial variance of the scalp field measurements, which is identical to an analysis of the Global Field Power (GFP) [6, 7] of the data. GFP analyses are currently not implemented in the software but will follow.

### 2.4. Visualization of Scalp Field Differences

Mean scalp field differences between two conditions or groups can easily be displayed using difference maps. If more than two conditions need to be compared simultaneously, this gets, however, increasingly complex, because the differences between all possible pairs all may have a different spatial distribution. A classical way to deal with such problems is multidimensional scaling (MDS) that allows to downscale high-dimensional result spaces into lower dimensional ones that can be easier visualized. Based on a matrix of similarities among all observations, multidimensional scaling represents each observation as a point in a lower-dimensional space, such that the closeness of the observation points optimally represents the original similarities. 

In the current case, the number of sensors defines the original amount of dimensions of the result space; this has to be reduced to a two-dimensional space in order to be displayed on a computer screen. The similarities between the mean scalp fields of the different conditions and/or groups can be assessed using the covariance between these maps. In this case, the two-dimensional space that optimally represents the entire matrix of covariances is spanned between the first two eigenvectors obtained from this covariance matrix [10, 11]. For the purpose of visualization, each mean scalp field is projected onto these two eigenvectors, which yields the two-dimensional coordinates of each mean different scalp field in this optimized two-dimensional result space. These coordinates of the different scalp fields are then displayed as points in a scatterplot. If two points are relatively close, this indicates that the corresponding scalp fields were relatively similar; if two points are relatively far apart, the scalp fields were relatively different. The direction of the difference between two points in the scatterplot and the scalp distribution of the firsts and second eigenvectors furthermore give an approximate account of the distribution of the scalp field difference between the two corresponding scalp field distributions.

## 3. Implementation

The program presented here implements the above described statistical procedures for the statistical comparison of event-related EEG and MEG multichannel scalp field data across a broad range of experimental designs. It is called Ragu (RAndomization Graphical User interface), making an allegation to the preparation of a ragout. A good ragout is obtained by slowly cooking many different ingredients until they are undistinguishable; this cooking is similar to the programs' procedure of increasing the data's entropy by randomizing until its constituents form an unstructured mixture. 

The program offers the possibility to compute these statistics either time point by time point, or on data averaged over some specified time interval. If time point by time point statistics are used, it further offers to compute the above-described overall statistics that prevent problems of multiple testing across time. Once the randomization statistics have been computed, the program displays all the effects (main effects and interactions) as line graphs showing the probability *P* of the null hypothesis as a function of time. If duration threshold statistics have been applied, periods of significance exceeding the critical duration are additionally marked. Results can then be interactively explored by displaying the mean scalp fields belonging to those within or between factors that constitute an effect. Additionally, these mean that scalp fields are displayed using multidimensional scaling.

Apart from the procedures described above, Ragu serves as a platform for the implementation of further statistical tools, such as microstate statistics. However, since these methods still await validation, an independent review, and publication, they are not further discussed here.

Ragu was developed under Matlab (http://www.mathwork.com/) and Windows 7. The program is available in the form of a downloadable standalone Matlab graphical user interface compiled for Windows using MS Visual Studio 2005; a Matlab license is therefore not necessary. The source code can be made available upon request, and the Matlab background should ensure cross-platform portability. The program is freeware; we attempt, but do not guarantee, support for bugs and questions that are not obvious from the manual or the papers. We, however, request users who publish results based on the output of the program to quote some of the conceptual papers [2–4] or the current paper. 

The program uses standard, plain text-based ASCII input files that contain time *x* sensor matrices of scalp field potentials. This should avoid problems of incompatible data format but offers little control over possible mistakes in channel sequences and so forth. Checking the correctness of the imported data is therefore part of the user's responsibilities.

The program allows saving and loading previously imported data, definitions of designs, and obtained results. The program always saves the entire information to standard Matlab files: The data, the analysis parameters, and the results are thus always within the same container, ruling out uncertainties about what results have been obtained with what data and parameters. These files contain a structure with all the information used by the program. Users with Matlab skills can open these files in Matlab (V7.10 or above) and extract or modify the data according to their needs, but care must be taken not to corrupt the internal consistency of the information or false results may be obtained. Furthermore, Ragu can save and load Matlab figures files (V6.0 or higher); users with Matlab skills can thus use these figure files as basis for their figures. Output to metafiles and bitmaps is also available, as well as a tab-delimited text output to be used with spreadsheet applications.

## 4. Usage and Sample Analysis

### 4.1. Installation and Update

The Ragu installation package can be downloaded at http://www.thomaskoenig.ch/Ragu_pkg.exe. This package contains the Ragu program, the installer of the required Matlab runtime library, and a history of the changes made to the program across time. The installation of the runtime library is necessary only once, to later install newer versions of Ragu, it is sufficient to download and run the file http://www.thomaskoenig.ch/Ragu_pkg_NoMCR.exe, that is much smaller. Upon request to the first author, users can be put on a mailing list that alerts you whenever a new version of the program has been compiled and uploaded. The source code is also made available upon request.

### 4.2. Sample Data

As an example, Ragu has been applied on a dataset from Stein et al. [12]. For this experiment, 16 healthy English speaking exchange students living in Switzerland for the duration of one year were recruited. Subjects were recorded twice, once at the beginning of their stay when they had basic German language skills (day 1) and in the middle of their stay with improved German language skills (day 2). During the experiment, they read German sentences with either semantically correct (The wheel is ROUND) or false (The garden is SHY) endings. It is known from previous studies that the violation of the semantic expectancy generated by the first part of the sentence (The garden is) produces an ERP scalp field called N400 in response to the last word (SHY) which is proportional to the degree of violation of the individual semantic expectancy [13].

The data analyzed here consists thus of four conditions: sentences with correct endings at day 1, with false endings at day 1, and with correct and false sentence endings at day 2. This represents a two-factorial design consisting of the factors “day” (day 1 and day 2) and “expectancy” (correct or false). The EPRs were recorded from 74 scalp locations with a 250 Hz sampling rate, were low-pass filtered at 8 Hz, and lasted from the onset of the last sentence word to 1000 ms after stimulus. Additionally, all subjects performed language tests at day 1 and day 2; thus, an overall score of language proficiency increase from day 1 to day 2 was available.

### 4.3. Data Import

Ragu stores all scalp field data to be analyzed internally in a single four-dimensional matrix (number of subjects *x* number of conditions *x* sensors *x* time points). To import data, the user has to provide, for each condition and subject, a plain ASCII file with only the measurements, one row for each time point, and one column for each sensor. The naming of the files has to be such that each file contains a tag that is unique for each subject, and a tag that is unique for each condition; the remaining parts of the filename must be identical for all files. All files have to be in the same directory (also if there are several groups of subjects), and missing data is not allowed. 

**Figure 1:**
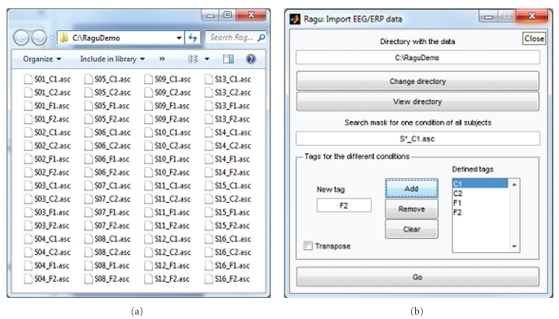
Ragu data import. (a) shows the directory containing data to be imported. The first 3 characters code the subject (“S01”, “S02”,…), characters 6 and 7 (“C1”, “C2”, “F1”, and F2) code for the 4 conditions. The dialog (invoked by Data -> Import command) with the parameters to import the data is shown on the right side. With the provided search mask, a list of the expected file names of one condition and all subjects is constructed (note that the search mask must find exactly one among all conditions). This list is then extended to the remaining conditions using the specified tags. Thus, as a crosscheck, one of the tags for the conditions has to appear somewhere in the search mask. Then, all the data is read based on the expected file names. If the importing of the data was successful, a confirmation is given informing the user about the dimensions of the imported data matrix. Otherwise, the output window displays error messages that may help identifying the problem.

According to Figure 1, in our example, we search the different files with S*_C1.asc and define 4 conditions: C1 (correct sentence ending day one), C2 (correct sentence ending day two), F1 (false sentence ending day one), and F2 (false sentence ending day two).

Once the data have been successfully imported, one can optionally specify further parameters such as the sampling rate and the latency of the event onset, and the montage (the possible formats are simple and specified in the online help), which helps for the later interpretation of the results. After the data and its additional parameters have been defined, it is recommended to briefly verify with the View->View data command whether the program represents the data as expected.

### 4.4. Defining and Understanding Within-Subject Designs

**Figure 2:**
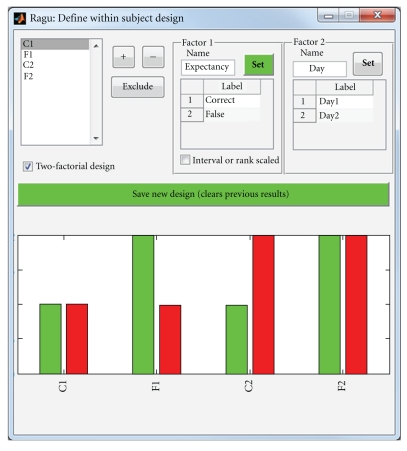
Specification of the within-subject design of the sample analysis in Ragu. In the upper left list box, all conditions are shown. For each factor, the levels of a condition can be set with the + or − button. To choose which factor to define, the “Set” buttons of the two possible factors are used. Additionally, the factors and factor levels can be labelled, and conditions can be excluded.

The experimental design is specified separately for within- and between-subject factors. Within subjects, it is possible to define up to two factors, and each factor can have several levels. If two factors are defined, the levels of the two factors must be orthogonal.  Figure 2 shows the dialog where users can enter the within-subject design of their experiment (invoked by Design->Within Subject Design). Users can name each factor and assign a label to each level of each factor, which will help for the later interpretation of results using multidimensional scaling. It is also possible to exclude some conditions from the analysis for post-hoc comparisons.

As visible in Figure 2 and introduced before, our sample consists of the factor “expectancy” containing the two levels “correct” and “false” and the factor “day” with the levels day 1 and day 2.

Once all the data has been imported, the data parameters have been set, and the within-subject design has been defined, the program is ready to compute the corresponding TANOVA. For these computations, a number of options are available (Analysis->Randomization options). 

Most importantly, and as discussed above, it can be specified if and how the data is normalized before the statistics are computed. If the L2 norm of the raw data is chosen, each individual scalp field of each condition is scaled to unity variance. This is the recommended type of normalization. For backward compatibility, it is also possible to normalize on the level of group/condition grand means; this is invoked by choosing dissimilarity [7] for normalization. 

Furthermore, the number of randomization runs can be chosen. The recommended number for an accurate estimate of the significance at the 5% level is 1000 runs, for the 1% level, it is 5000 runs [8], but as the computation of so many runs is lengthy, lower number can also be sufficiently informative for exploratory purposes.

Finally, it is possible to adjust the threshold for the acceptance of significance; this affects the display of results and the statistics on temporal cluster-size thresholds.

**Figure 3:**
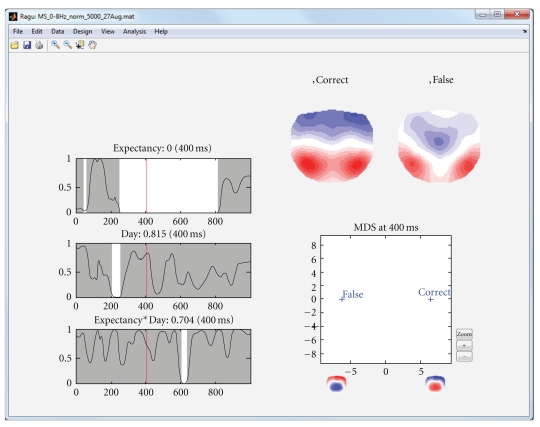
Display of the results of the analysis of the within-subject factors in the sample dataset. The left part of the display shows the significance of the TANOVA's main effects (expectancy and day) and their interaction as line graphs showing the probability *P* (*y*-axis) of the null hypothesis as a function of time (*x*-axis). Significant time points (*P* < .05) are marked in white. By clicking in a graph, a cursor is set, the *P* value of the respective time point is displayed besides the graph title, and the effect is mapped on the right side of the display. In the current figure, the main effect of expectancy (correct versus false sentence endings) at 400 ms is displayed. The upper right part of the display shows the mean topographic maps of all factor levels from the graph where the cursor has been set. In the present display of the main effect of expectancy, these are the mean maps across subjects and days of correct and false sentence endings at 400 ms. For the figure in the lower right part, these two mean maps have been fed into an MDS analysis. For this purpose, all mean maps were submitted to a spatial PCA. The *x*-axis of the figure represents the projection of mean maps onto the first eigenvector. The spatial distribution of the eigenvector is represented by two topographic maps below the *x*-axis. The graph indicates that the “false” condition is more negative and the correct condition is more positive at parietal electrodes.

The first analysis of the sample data that we presented above is based on a purely within-subject design; all subjects are expected to show comparable effects, and no between-subject factor has been defined. After running a TANOVA with this design, the program displays a graph with the significance of each within-subject factor as a function of time (main effects), and the interactions of the factors (Figure 3, left part). In the case of our sample dataset, we therefore obtain a main effect of day, a main effect of expectancy, and an interaction of expectancy and day. The user can click into these graphs; this will display the obtained *P* values at the selected time period. In addition, the mean scalp field distributions of all groups and factor levels that form part of the effect are shown (Figure 3, right part). Finally, those mean scalp field distributions are submitted to a multidimensional scaling and projected upon the first two resulting eigenvectors. Those projections are shown in a scatterplot as shown in Figure 3 (lower right part). This scatter plot allows an intuitive first interpretation of an effect; the further two points are apart, the larger the difference among the corresponding mean maps is. Figure 3 illustrates the display based on the sample data.

### 4.5. Defining and Understanding Between-Subject Designs

Apart from being able to investigate up to two within-subject factors, it is also possible to define a between-subject design to run analyses that account for individual or group differences. When invoking the between-subject design dialog (Design->Between Subject Design), the program lists the data files of all subjects of one (arbitrary) condition, and the user can assign each subject to a specific group. Alternatively, when checking the “Continuous/rank data” box, each subject can be assigned an individual value that quantifies some interindividual factor. This factor has to be interval or rank scaled and will be considered as covariate for a TANCOVA [4]. If necessary, individual subjects can also be excluded from the analysis.

**Figure 4:**
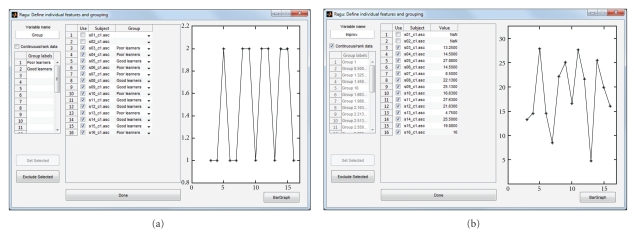
Specification of the between-subject design of the sample analysis in Ragu. This figure shows the mask for the definition of the between-group design. The variable name appears later on the output of the results. As seen in both examples, no behavioural measures exist for subjects 1 and 2. These subjects are excluded from the analyses by unchecking the “use” checkbox. The line graphs on the right of each example show the value filled in for each subject. (a) The division of subjects in a group of low language proficiency improvement and a group of high language proficiency improvement. The values 1 or 2 are given to each subject as shown in the line graph. (b) For the computation of a TANCOVA, the “continuous/rank data” box has to be checked. Then, the value of proficiency increase from day one to day two can be entered individually. The line graph shows the level of increase of each subject.

**Figure 5:**
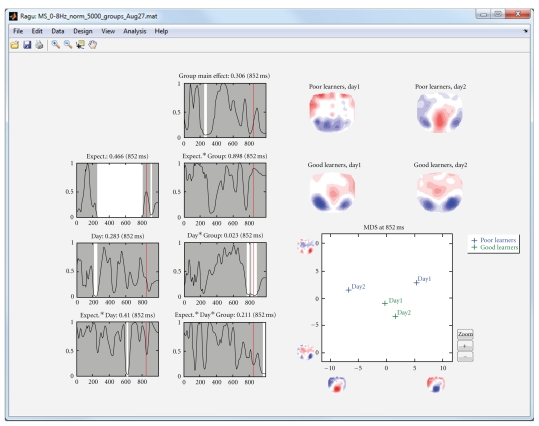
Display of the results of the analysis of the sample dataset when subjects were divided into two groups with low and high language improvement. The left-most row of line graphs shows the same information as the line graphs of Figure 3 (except for 2 less subjects); the additional line-graphs show all effects including the new factor group. The interaction day by group shows a significant effect around 850 ms. Because there are more than two mean maps, the MDS figure now also contains a *y*-axis that shows the projection of the mean maps onto the second eigenvector. The MDS indicates that the day by group interaction is mainly due to the differences between days 1 and 2 in poor learners; good learners show a much smaller change. The result suggests that from day 1 to day 2, poor learners have changed in late, feedback-related processing, while good learners have maintained their initial processing strategies. The same conclusion is also deducible from the mean maps shown in the upper right part of the display.

In the following analysis of the sample data, we divided the subjects into a group with above median German proficiency increase from day 1 to day 2 (“good learners”) and a group with below median German proficiency increase (Figure 4(a)). When computing a TANOVA, in addition to the effects already known from the pure within-subject analysis (Figure 3), we obtain the interaction of group membership with day, the interaction of group membership with expectancy, and the triple interaction of group membership, day, and expectancy (Figure 5).

The output of this group ANOVA shows an effect of day*group in a late time interval around 800 ms. 

Alternatively, instead of subdividing the subjects into groups based on their performance, it is possible to investigate whether there is evidence for components that are linearly related to performance across subjects. This approach is called TANCOVA and is also available in the program. By checking the “continuous/rank data” box in the between-subject design dialog, the individual performance (learning rates in the present example) can be entered (Figure 4(b)), and the program will compute a TANCOVA.

**Figure 6:**
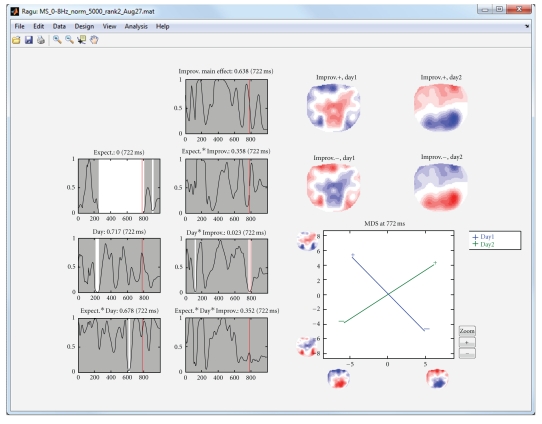
TANCOVA of the ERPs with the increase of language proficiency. The second row of graphs shows the same information as in Figure 5, with the exception that the effect of language improvement has been taken into account as a linear predictor. Again, the interaction day by improvement shows a late effect now somewhat before 800 ms. The mapping of this interaction on the right side shows the positive and negative covariance maps separately for days 1 and 2. These covariance maps are again entered into an MDS. It appears that there is an almost an orthogonal relation among the language improvement and ERP topography on day 1 and day 2 at this time range.

In our sample, we investigated whether the ERPs at day 1 have a predictive value for the increase in language proficiency from day 1 to day 2.  Figure 6 shows the results of computing moment-by-moment TANCOVAs of the ERPs with the increase of language proficiency.

### 4.6. Further Statistics

**Figure 7:**
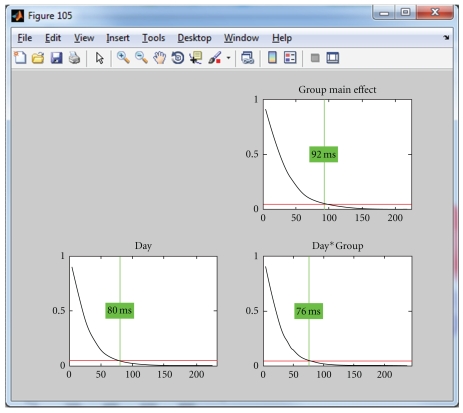
Estimation of a duration threshold of significant TANOVA effects. The threshold is estimated for each effect separately. The horizontal axis indicates the duration of continuous epochs with local significances of the TANOVAs below the selected threshold. The vertical axis indicates the probability of encountering a certain effect duration under the null hypothesis. These durations are obtained by “testing” the results of the randomization runs against each other [2, 3]. The red line indicates the chosen threshold for overall significance, in the current case, *P* = .05. The vertical green line indicates the duration that is longer than (1 − *P*) percent (in the present case 95%) of all randomly obtained effect durations under the null hypothesis. This threshold can then be applied to the previously obtained TANOVA results. Thresholds have been computed based on the result of the time point-wise sample TANOVA analysis of Figure 8.

**Figure 8:**
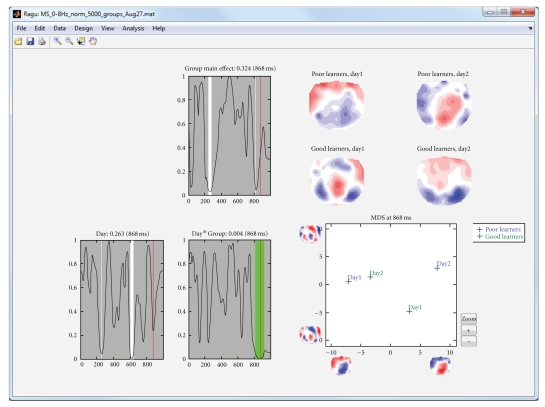
Results of a TANOVA computed for correct sentence ending on days 1 and 2, with good and poor learners as groups. The duration threshold estimate from Figure 7 has been applied. Periods meeting the duration criterion are shown in green. The late group by day interaction meets the duration criterion, whereas the other effects do not.

Using a global measure of differences across all channels eliminates the problem of multiple testing across sensors, but since the previous analyses have been conducted time point by time point, false positive results may have been obtained due to multiple testing across time. To protect against these, it is possible to compute statistics on the overall count of significant time points and the duration of significant effects as discussed above.  Figure 7 shows and explains how such overall thresholds can be obtained for the duration of continuous periods of significance of the time point by time point analyses. For the simplicity of the example, we used only the correct sentence endings in this analysis. In Figure 8, the obtained duration threshold has been applied to the TANOVA results. 

**Figure 9:**
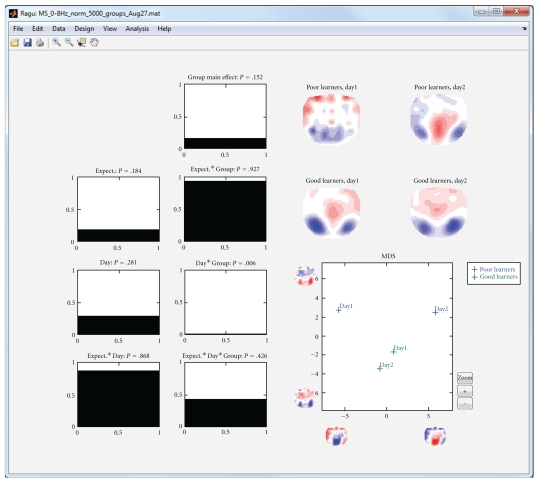
Group TANOVA over averaged time points between 780 and 890 ms. When averaged across multiple time points, the graphs on the left each shows significance levels for the whole averaged time span as bar graphs instead of line graphs. The *y*-axis shows again the level of significance (*P*), which is now written above each graph for the whole time span.

If there is an a-priori hypothesis about a time window where some effect should be tested, one can also compute the analyses outlined above based on topographies averaged across a time interval. As an example, we took the results of the group analysis with the factors day, expectancy, and group as described above. Based on the results of the cluster duration test (Figure 8), we wanted to know whether the effect is consistent across time points and stable if we average the signal over the time points between 780 and 890 ms. We thus computed the TANOVA again over the averaged time frame. The results are displayed in Figure 9. They show that the effect is indeed stable across time points as it persists when averaging. 

In addition, and independently of comparisons among groups and conditions, the program contains a module to compute the topographic consistency test (TCT, [2]) that assesses, for each group and condition, during which time points there is evidence for a consistent pattern of active sources across subjects (invoked with Analysis->Topographic Consistency Check); a detailed explanation of this method is found in [2]. This test can optionally be computed at the beginning of the analysis to define the analysis window.

**Figure 10:**
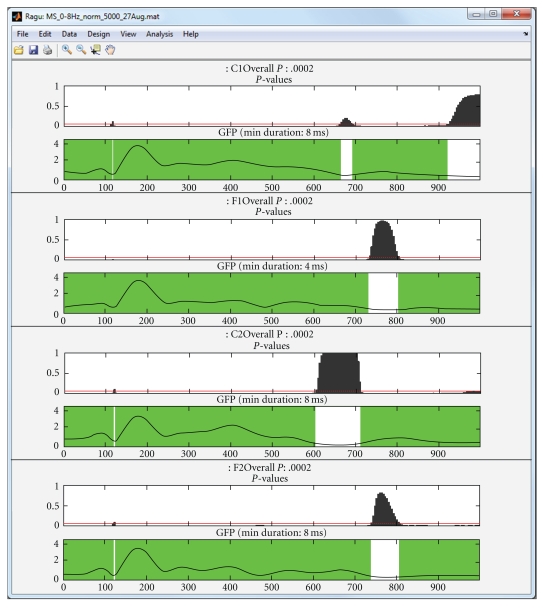
Topographic consistency test (TCT) applied to the four within conditions of the sample data (C1: correct sentence ending day 1; F1: false sentence ending day 1; C2: correct ending day 2; F2: false ending day 2). For each condition, two graphs are shown. The upper one displays the *P* value obtained by the TCT (vertical axis) and the chosen threshold (red line). The *x*-axis displays the time from 0 to 1000 ms from the word onset on. The second graph shows the Global Field Power (GFP), with periods of consistent topographies marked in green. The *y*-axes of the lower graphs indicate the GFP amplitude in *μ*V.

As Figure 10 shows, there is evidence for common activations across subjects over prolonged time periods. The first period of consistent topography lasts until about 600 to 700 ms and continues after an interruption until the end of the data. It is also evident that the significance level of the test is inversely related to the GFP of the ERP.

### 4.7. Summary of Results

The results of our sample analysis showed an interaction effect of expectancy*day from 600 to 650 ms, mainly due to the difference of topographies of correct word endings from day 1 to day 2. Additionally, in the group analysis, an interaction effect day*group from around 780 to 890 ms was seen. This interaction effect was mainly due to the change of topographies from day 1 to day 2 in the group of bad learners. Since we saw that the interaction expectancy*day was due to differences in correct word endings, we assumed that this may also play a role in the interaction effect day*group. Thus, bad learners should show a change of processing of correct words from day 1 to day 2. 

We tested our assumption as formulated above in a new design with the factors group and day, with day containing only correct sentence endings at day 1 and day 2. This TANOVA resulted in a more stable interaction effect in the same time frame as indicated by the cluster size test. Computing this new TANOVA again averaged over the important time frame resulted in a significant interaction group*day, indicating that the effect is stable and consistent over the respective time points.

Finally, the consistent topography test supported our results showing that the processing duration of correct sentence endings was shorter at day 2 than at day 1, whereas the duration of the consistent topography did not differ between false sentence endings on day 1 and day 2.

This sample highlights the advantage of an analysis without the need of a-priori decisions. With a-priori choices we would have limited the analysis to search effects around 400 ms due to previous studies reporting about the N400 effect. We would have missed the results found around 800 ms mainly due to different topographies in response to correct sentence endings.

## 5. Discussion

In the current paper, we present software designed to compute statistical analyses on scalp field data using methods and algorithms based on randomization techniques that are custom tailored to the specific properties and problems of such data. The methods, user interface, and display of the results implemented in the program should accommodate most of the experimental designs that maintain an acceptable degree of complexity (two within-subject factors with multiple levels each, and one between-subject factor, also with multiple levels). The paper is thought as an introduction for researchers using EEG/MEG data that want to understand the basic concepts of the methods and make use of the software. For a more thorough discussion of the underlying concepts, we refer to other publications [2–4]. 

In terms of the “flow” of an analysis of event-related scalp field data, the methods and tools presented here offer a good starting point, but typically not the end point of an exhaustive analysis of a data set. The main advantage of beginning an analysis with the methods proposed here is that they offer robust, powerful, and physiologically meaningful statistics on the entire, untransformed, and unbiased set of measurements. Thus, without the need to select sensors, time windows of interest, type and parameters of inverse solutions, or other a-priori choices, the data informs the researcher about whether and when there is a significant effect of some experimental manipulation. At the same time, significance indicates that the conditions and groups involved in the effect activated at least partially different sources and thus assumingly different brain functions. Once such a global statistical basis has been established, the data can be further manipulated to be explored more locally in sensor or inverse space. In other words, we hope that the methods and tools introduced here can help to minimize the dependence of statistical evidence from a-priori choices of specific models.

A further remark to be made here is on the general difference of assumptions when doing statistics on the scalp compared to the source level. Consistent scalp fields indicate consistent source localization and source orientation, while source orientation is typically not considered in voxel-wise statistics of inverse solutions. As argued before [3], source orientation appears to be a very robust and sensitive feature of ERP data; all results obtained by averaging evoked scalp potentials imply that not only the amplitude of the sources of the evoked potential was constant, but also their orientation. The interpretation of what a consistent change of orientation means remains less clear. On the other hand, statistics based on inverse solutions obviously depend on the correctness of the assumptions of the inverse model. Furthermore, at least for distributed inverse solutions, the result space is drastically inflated without an increase of degrees of freedom of the data, and heavy corrections for multiple testing across voxels need then to be applied post-hoc to correct for this somewhat artificial problem. 

A disadvantage of the program (common to all programs that are based on randomization and resampling techniques) is that computation time increases linearly with the amount of randomization runs, which can make computation time lengthy for larger datasets. In its current implementation, the program runs as a single-thread process, such that it creates limited interference with performance when running in the background. Parallelization is, however, planned in future releases. 

Another limitation is that the program is academic software and under constant development. Since there are no separate alpha and beta releases, it may contain undocumented, more experimental options that are not yet meant for the general public (e.g., analyses in the frequency domain). So, unless you do not know precisely what to expect, please do not use them. And finally, the program has been developed and is maintained with limited resources; careful crosschecking of the plausibility of the results is mandatory; user support may become limited, but suggestions, problem reports, and criticism are always welcome.
